# Respiration data on sleep state misperception, psychophysiological insomnia and normal individuals from a cross sectional study

**DOI:** 10.1016/j.dib.2019.104428

**Published:** 2019-08-22

**Authors:** Mohammad Rezaei, Behnam Khaledi Paveh, Soroush Maazinezhad, Habibolah Khazaie

**Affiliations:** a Sleep Disorders Research Center, Kermanshah University of Medical Sciences, Kermanshah, Iran; b Department of Psychiatric Nursing, School of Nursing and Midwifery, Kermanshah University of Medical Sciences, Kermanshah, Iran

**Keywords:** Sleep dataset, Sleep state misperception, Paradoxical/psychophysiological insomnia, Respiratory, Breathing

## Abstract

The data prepared here had been originally collected for a study project entitled ‘Breathing pattern analysis in insomnia suffers’. This data describes the information of 82 individuals; participating 41 normal individuals and 41 insomnia suffers with tow phenotype included 30 sleep state misperception and 11 psychophysiological suffers. The data presents 8 hours of respiratory signals included flow pressure, flow temperature, Oxygen saturation, Thorax and Abdomen signal in frequency sampling 256, 32, 32, 32, 4 Hz respectively. It includes breathing features and sleep profiles in segments of 30s for each individuals. In addition, the full demographic and objective specifications was attached.

**Table:** Specifications Table

Subject	Psychiatry, Sleep disorder
Specific subject area	Respiratory, insomnia, Sleep
Type of data	Table, text file, matlab file
How data was acquired	Polysomnography, Matlab software
Data format	Filtered, analyzed, subjective and objective data are collected in xlsx excel, txt and mat formats
Parameters for data collection	Subjective parameters included Age, gender, height, weight, education, marriage, and body mass index Objective parameters included sleep profile, SpO2 events, flow events, respiratory factors.
Description of data collection	Subjective parameters was collected through quaternary and interview. For collection of objective parameters, Polysomnography instrument was also used.
Data source location	Institution: Sleep Disorders Research Center in Kermanshah University of Medical Science City/Town/Region: Kermanshah Country: Iran Latitude and longitude: 34°19′52.8″N 47°03′24.7″E
Data accessibility	*The dataset is freely available at*[1]*for any academic, educational, and research purposes.* Repository name: Mendeley Data Data identification number: https://doi.org/10.17632/zt4rgyf5yf.2 Direct URL to data: https://data.mendeley.com/datasets/zt4rgyf5yf/2

**Table:** 

**Value of the data** • The data includes information that is both raw and analyzed which could be utilized in other studies. • The data included 8 hours of respiratory signals during sleep bouts (flow pressure, flow temperature, SpO2, Thorax, Abdomen) from 82 individuals; participating 41 normal individuals and 41 insomnia suffers with tow phenotype included 30 sleep state misperception and 11 psychophysiological suffers. • Respiratory analysis and investigating role of oxygen in each stage of sleep and brain function is important. • The data can likewise be utilized to assess the pattern of breathing in sleep state misperception and psychophysiological suffers. • The diagnosis was subjectively and objectively performed by a sleep specialist, based on sleep highlights.

## 1 Data

**Fig. 1:**
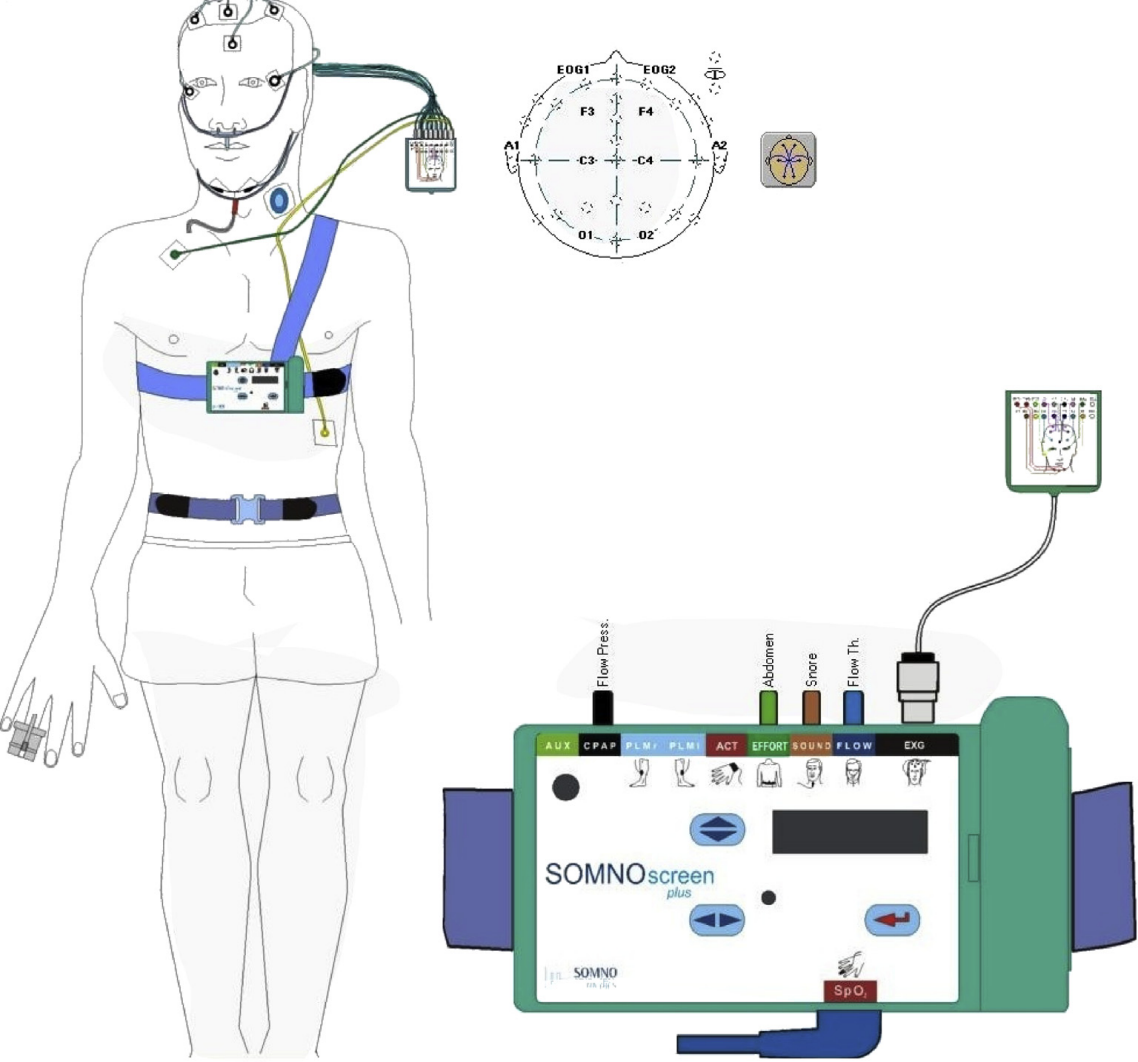
Sensors montage included flow pressure, flow temperature, Spo2, Thorax, and Abdominal.

**Fig. 2:**
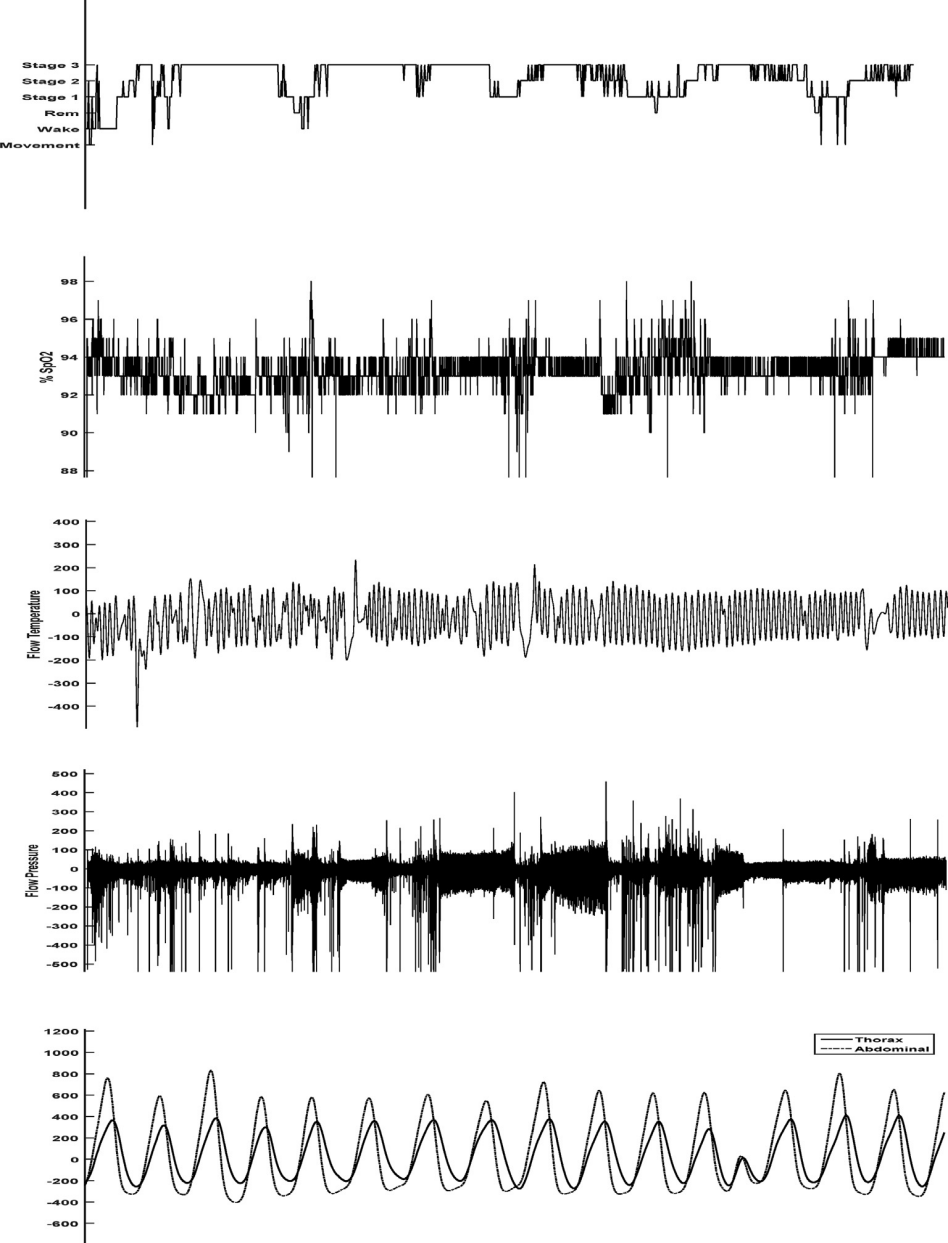
A sample show of respiration signals included sleep profile, SpO2, flow temperature, flow pressure, thorax, and abdominal.

The data of this article provides information about the cross-sectional study entitled “Breathing pattern analysis in insomnia suffers”. All of the data prepared in three category including table, text, and matrices and arrays indexed in Matlab. Demographic information, Subjective sleep parameters, objective sleep parameters such as apnea-hypopnea index, minimal and average SpO2, present of sleep stages, TST and %SE presented in an Excel file. Sleep profiles and breathing features such as flow events and SpO2 events presented in segments of 30s for each individual during sleep as text file. Respiratory signals of all participants were collected in matrices and arrays indexed in Matlab as mat format. The data presents 8 hours of respiratory signals included flow pressure, flow temperature, Oxygen saturation, thorax and abdomen signal in frequency sampling 256, 32, 32, 32, 4 Hz respectively (Fig. 1, Fig. 2). It should be noted that the respiratory signals were filtered other than SpO2 using the low pass filter of 1 Hz by SOMNOscreenTM plus device. Signals were arranged in the form of structure array. They were named as X_XX.mat in which 'X' represents individuals subtype such as normal, sleep state misperception suffers and psychophysiological insomniacs and so 'XX' represents the name of signal such as PressureFlow, FlowTh, SpO2, Thorax and Abdom. The names of data and their descriptions was listed in Table 1. The dataset is freely available at [1] for any academic, educational, and research purposes.

**Table 1:** The name of the recorded information and their description.

Name	Description
Demographics_Subjective_Objective information.xlsx	This is an Excel file in which was expressed the demographics, and Subjective and Objective information for all of individuals.
Flow Evets.txt	The events that occur to drive air flow into the lungs
SpO2 Events.txt	The occurred Oxygen desaturation events
SpO2.txt	The Oxygen saturation rate during sleep in epochs of 30s
Sleep Profile Reliability.txt	Reliability of the sleep profile
Sleep Profile.txt	Time of sleep stages including Stage 4, Stage 3, Stage 2, Stage 1,Rem,Wake, Movement in epochs of 30s
PressureFlow.rar	Flow pressure signal for all of participants in sampling rate 256 Hz
FlowTh.rar	Flow temperature signal for all of participants in sampling rate 32 Hz
SPO2.rar	Oxygen saturation rate for all of participants in sampling rate 4 Hz
Abdom.rar	Abdominal signal for all of participants in sampling rate 32 Hz
Thorax.rar	Thorax signal for all of participants in sampling rate 32 Hz

## 2 Experimental design, materials and methods

### 2.1 Sample collection

There were the 82 individuals from 16 to 64 years of age (39.77(13.40)) recruited for participating included 39 males (47.56%) and 43 females (52.44%). Thirty and eleven individuals suffered respectively from sleep state misperception and psychophysiological insomnia among the participants. Other individuals as normal sleeper were chosen from general people. Initially, normal sleepers were chosen based on Pittsburgh questionnaire. Finally, we selected normal sleeper who were normal according to the result of PSG (Table 2).

**Table 2:** Demographic information included gender distribution, age and BMI between normal sleepers and insomnia groups.

	Normal (N = 41)	Insomnia (N = 41)	
		Sleep state misperception (N = 30)	Psycophysiological insomnia (N = 11)
Age	37.88 (13.89)	4.10 (9.96)	44.00 (13.27)
Sex			
Female	14(34.15%)	20(66.67%)	9(81.88%)
Male	27(65.85%)	10(33.33%)	2(18.18%)
BMI	26.21 (4.96)	26.22 (3.45)	26.60 (3.71)

### 2.2 Procedure

Invited participants to sleep laboratory recommended avoiding coffee, tea, heavy diet and a cigarette. Using clinical interview by a sleep specialist according to ICSD II [2], insomnia suffers selected initially. In addition, normal sleeper were also interviewed to exclude any sleep disorders. All of participants completed the demographic and subjective questionnaire in Persian version to measure subjective sleep characteristics. The Pittsburgh questionnaire was used for this purpose. PSQI is a self - report questionnaire evaluating subjective sleep over an interval of 1 month. The measure is made up of seven components to calculate a global score as sleep quality index [3]. Next, PSG test used to objective data acquisition. The test duration was about 8 hours. Polysomnography device called SOMNOscreenTM plus PSG manufactured by SOMNOmedics GmbH, Germany. Finally, based on both subjective and objective information data, insomnia diagnosis was done. PSG room has been standardized for audial and visual stimulation and any artifact according to international standards [4].

## Ethical approval

All procedures performed in the data collection involving human participants were in accordance with the ethical standards of the Ethical Research Committee of Kermanshah University of Medical Sciences, and with the 1964 Helsinki declaration and its later amendments or comparable ethical standards. In addition, informed consent was obtained from all individual participants.
